# Chronic Obstructive Pulmonary Disease Patients Have Increased Levels of Plasma Inflammatory Mediators Reported Upregulated in Severe COVID-19

**DOI:** 10.3389/fimmu.2021.678661

**Published:** 2021-07-15

**Authors:** Nathalie Acevedo, Jose Miguel Escamilla-Gil, Héctor Espinoza, Ronald Regino, Jonathan Ramírez, Lucila Florez de Arco, Rodolfo Dennis, Carlos A. Torres-Duque, Luis Caraballo

**Affiliations:** ^1^ Institute for Immunological Research, University of Cartagena, Cartagena, Colombia; ^2^ Informatic Unit, INMEDIT SAS and Faculty of Engineering, University of Cartagena, Cartagena, Colombia; ^3^ Clinica Respiratoria y de Alergias, Cartagena, Colombia; ^4^ Departamento de Investigaciones, Fundación Cardioinfantil, Bogotá, Colombia; ^5^ Research Department - CINEUMO, Fundación Neumológica Colombiana, Bogotá, Colombia; ^6^ Research Department and Specialization Program in Pulmonology, Universidad de la Sabana, Bogotá, Colombia

**Keywords:** COPD, CXCL9, HGF, IL6, severe COVID-19, plasma proteomics

## Abstract

**Background:**

Chronic obstructive pulmonary disease (COPD) is associated with increased risk of severe COVID-19, but the mechanisms are unclear. Besides, patients with severe COVID-19 have been reported to have increased levels of several immune mediators.

**Methods:**

Ninety-two proteins were quantified in 315 plasma samples from 118 asthmatics, 99 COPD patients and 98 healthy controls (age 40-90 years), who were recruited in Colombia before the COVID-19 pandemic. Protein levels were compared between each disease group and healthy controls. Significant proteins were compared to the gene signatures of SARS-CoV-2 infection reported in the “COVID-19 Drug and Gene Set Library” and with experimentally tested protein biomarkers of severe COVID-19.

**Results:**

Forty-one plasma proteins showed differences between patients and controls. Asthmatic patients have increased levels in IL-6 while COPD patients have a broader systemic inflammatory dysregulation driven by HGF, OPG, and several chemokines (CXCL9, CXCL10, CXCL11, CX3CL1, CXCL1, MCP-3, MCP-4, CCL3, CCL4 and CCL11). These proteins are involved in chemokine signaling pathways related with response to viral infections and some, were found up-regulated upon SARS-CoV-2 experimental infection of Calu-3 cells as reported in the COVID-19 Related Gene Sets database. An increase of HPG, CXCL9, CXCL10, IL-6, MCP-3, TNF and EN-RAGE has also been experimentally detected in patients with severe COVID-19.

**Conclusions:**

COPD patients have altered levels of plasma proteins that have been reported increased in patients with severe COVID-19. Our study suggests that COPD patients have a systemic dysregulation in chemokine networks (including HGF and CXCL9) that could make them more susceptible to severe COVID-19. Also, that IL-6 levels are increased in some asthmatic patients (especially in females) and this may influence their response to COVID-19. The findings in this study depict a novel panel of inflammatory plasma proteins in COPD patients that may potentially associate with increased susceptibility to severe COVID-19 and might be useful as a biomarker signature after future experimental validation.

## Introduction

During the COVID-19 pandemic, populations at risk of severe disease have been detected including patients with asthma and COPD (1). Indeed, COPD patients had a higher risk for intense care unit (ICU) admission, mechanical ventilation, or death, even after adjustment for age and smoking (2). A recent meta-analysis has reported that COPD is associated with a significant, over fivefold, risk of severe COVID‐19 (3). The risk has been attributed to the overexpression of angiotensin converting enzyme 2 (ACE-2) receptor in bronchial epithelial cells of COPD patients (4) and the increased expression of the transmembrane protease serine 2 (TMPRSS2) induced by the exposure to cigarette smoking (5). A recent study also showed that patients with chronic lung disease (including COPD) have changes in cell-type specific expression of genes related to viral replication and the immune response, that could promote immune exhaustion and altered inflammatory gene expression (6), supporting the increased risk in COPD patients. On the other hand, severe asthma is associated with COVID-19 related death (7) and asthmatic patients show increased expression of the viral activator TMPRSS2 (8). Although there is still controversy on whether asthmatics are more susceptible to get infected with SARS-CoV-2 (9), it is recognized that endotype and other comorbidities may increase risk to severe COVID-19 in some asthmatics (10).

There is also a growing interest in identifying protein biomarkers of severe COVID-19 that may allow to detect high risk patients for early management and to define point-of-care clinical classifiers (11–13). Several cohorts have described significant changes in protein biomarkers in patients with COVID-19. Initial studies revealed molecules that are indicative of inflammation (such as C reactive protein) or changes in cell proportions (neutrophil-to-lymphocyte ratio, NLR) (14, 15), but more recent researches are focused on elucidating pathways underlying patient severity with detailed profiling of plasma molecules such as cytokines, chemokines and growth factors (13, 16–18). Messner et al., identified 27 blood proteins associated with symptoms severity of COVID-19 patients, including complement, coagulation, inflammation modulators, and pro-inflammatory factors upstream and downstream interleukin 6 (19). In addition, Arunachalam et al., identified an increase in protein levels of MCP-3, TNF, EN-RAGE and TNFSF14, being higher in patients with severe disease and ICU admission (16).

Plasma profiling is a useful tool that can reflect inflammatory processes, including those in lung tissues (20). We implemented the multiplexed measurement of 92 plasma proteins to provide a comprehensive overview of protein levels in the participants of the “Identification of Biomarkers in Asthma and COPD (IBACO)” study. During the analyses we found that COPD patients show increased levels of several immune proteins that have been reported increased in patients with severe COVID-19. Then, we applied network analyses on the list of proteins detected in this study to evaluate their functional relationships, trying to understand how their dysregulation may influence the susceptibility of COPD patients to severe COVID-19.

## Materials And Methods

### Study Participants

This study was approved by the ethical committees of the University of Cartagena (nr. 4169722017) and the “Fundación Neumológica Colombiana” (nr. 232-07122017) and written informed consents were obtained from all participants. The plasma samples were obtained from the IBACO study; representative of adult patients from an urban setting in a middle-income country. Patients were recruited from two reference pneumology clinics of Cartagena and Bogotá. The study included a well characterized group of adult subjects aged 40 to 90 years with asthma (n=118) or COPD (n=99) recruited between February 2018 and March 2020, before cases of SARS-CoV-2 infection were reported in the country. Healthy controls (n=99) were recruited during the same period in elderly homes with a similar age and gender distribution. At the time of sampling subjects were queried about their current and past sociodemographic characteristics, symptoms, comorbidities, smoking habits, environmental exposures, history of allergies and pharmacological treatments. Physical examination and pulmonary function tests were performed to all participants. The diagnosis of asthma or COPD was done by a pulmonologist according to the GEMA guidelines for asthma (21) and to the GesEPOC guidelines for COPD (22). Pulmonary function was evaluated using spirometry pre- and post-bronchodilator according to the American Thoracic Society (ATS) guidelines. Quality of life was assessed by the asthma control questionnaire (ACQ-5), the COPD Assessment Test (CAT) and the Saint George’s Respiratory Questionnaire (SGRQ). Comorbidities were evaluated using the Charlson comorbidity index. Inclusion criteria were: 1) age of 40 years or more, 2) clinical diagnosis of asthma or COPD confirmed by a pneumologist (COPD was defined by a postbronchodilator forced expiratory volume in one second [FEV_1_]/forced vital capacity [FVC] ratio [FEV1/FVC] less than 0,7 plus a reported exposure to wood smoke for at least 10 years and/or cigarette smoking with more than 10 packs/year). Exclusion criteria were exacerbation of asthma or COPD in the last 8 weeks, presence of uncontrolled comorbidities such as hypertension, coronary disease, hepatic and/or renal diseases, active neoplastic disease, treatment with immunosuppressive drugs, human immunodeficiency virus (HIV) infection, report of respiratory or non-respiratory infection in the last 8 weeks and/or being under treatment with monoclonal antibodies.

### Sample Collection

Blood samples were collected by standard phlebotomy in heparinized tubes and plasma was separated by centrifuging at 1000 g at 4°C for 15 minutes and stored at -80°C until analysis. Another sample was collected in an EDTA tube to measure leukocyte cell counts by type IV hemocytometry. IgE antibodies were measured by ImmunoCAP following manufacturer instructions (Thermo Fisher, Uppsala, Sweden).

### Quantification of Plasma Proteins

For plasma profiling, the samples were randomly distributed in 96-well plates and protein levels were measured by the Proximity Extension Assay (PEA) (23) using the Target 96 Inflammation Panel (Olink Proteomics, Analysis Service Facility, Boston, USA) which includes a broad selection of proteins established as inflammatory signatures in diverse inflammatory diseases. A total of 67 out of 92 plasma molecules were detected in heparinized plasma (73%). Normalized Protein Levels were expressed as NPX units (log_2_ scale). Intraassay and interassay average coefficient of variations (%CV) were 6% and 11%, respectively. Nine samples were removed because they did not pass the quality control (QC). Twenty-five proteins had values below the limit of detection (LOD) and were removed from analyses.

### Statistical Analysis

Differences between protein levels among the study groups (asthma, COPD and healthy controls) were first screened by the F-test (ANOVA) using the Olink Insights Stat Analysis Web tool (https://olinkproteomics.shinyapps.io/OlinkInsightsStatAnalysis/) and by the non-parametric Kruskal Wallis test. Given that some of the proteins with significant differences between groups did not have a normal distribution (Kolmogorov-Smirnov test), we implemented both independent samples *t*-test and Mann-Witney tests for comparing protein levels between patients and controls. Correlation coefficients were calculated by the Pearson test. To adjust for multiple testing, the Benjamini–Hochberg false discovery rate (FDR) correction was applied using the p.adjust function. An adjusted *P* ≤ 0.05 was considered significant. The effect of age on protein levels was modelled by linear regression. Statistical analyses were performed in R version 3.5.3 (https://www.r-project.org/).

### Functional Annotation

The list of proteins with differences between patients and healthy controls were analyzed for pathways, ontologies, diseases/drugs in the Enrichr web tool (https://maayanlab.cloud/Enrichr/) (24). Enrichment on the Kyoto Encyclopedia of Genes and Genomes was evaluated in PathwAX II (https://pathwax.sbc.su.se/) and the bioconductor package clusterProfiler. The induced network analysis was constructed in Consensus PathDB (http://cpdb.molgen.mpg.de/) using an intermediate nodes z-score threshold of 30 and including binary protein-protein interactions of high confidence and biochemical reactions (25).

### Biomarkers of Severe COVID-19

We performed a literature search on all proteins experimentally measured and associated with severe COVID-19. We started with a set of 93831 articles downloaded on January 30^th^, 2021 from LitCovid, a curated open-source literature of Pubmed research papers related to COVID-19. Then we retrieved a corpus of documents on severe COVID-19 using the terms (“severe COVID” or “fatal COVID”) AND (“biomarker” OR “plasma marker” OR “plasma protein”). This resulted in 114 papers of which 20 reported associations of severe COVID-19 with 52 unique proteins. The protein list from this study was also compared to evaluate the enrichment with that reported in the COVID-19 Related Gene Sets (SARS 133 Literature-Associated Genes from Geneshot GeneRIF) and calculate the probability of detecting overlap due to chance.

## Results

### Characteristics of the Study Populations

**Figure 1:**
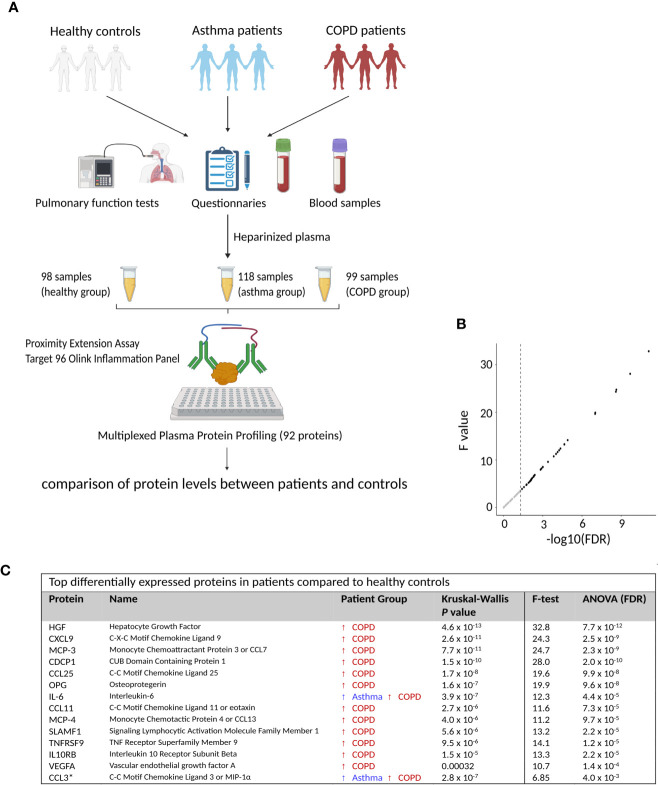
Identification of differentially expressed proteins in COPD. **(A)** Schematic representation of study groups and samples included in the proteomic screening. **(B)** Dot plot on the F value *versus* the False Discovery Rate (FDR) for all measured proteins. Dotted lines represent *P* = 0.05. **(C)** Top differentially expressed proteins by comparing asthma and COPD patients with healthy controls. Detailed information on protein names and *P* values is presented in **Supplementary Table 1**. This figure was drawn using BioRender.

Demographic and clinical characteristics of the study participants are presented in **Table 1**. As expected, asthmatic patients have higher IgE levels and eosinophils than healthy controls and COPD patients. On the other hand, COPD patients have higher numbers of neutrophils and monocytes while there were no differences in the number of lymphocytes. A total of 315 plasma samples were analysed with the Olink Inflammation panel to compare proteins levels between groups (**Figure 1A**).

**Table 1:** Demographic and clinical characteristics of study subjects.

Variables	Healthy controls (n=98)	Asthma patients (n=118)	COPD patients (n=99)	*P* value
Age, yr	56 ± 13	60 ± 11	72 ± 8	<0.0001
Female gender, n (%)	63 (64.3%)	82 (69.5%)	40 (40.4%)	<0.0001
BMI, kg/m^2^	24.9 ± 4.5	27.5 ± 4.9	25.1 ± 4.5	<0.0001
Tabaquism ever, yes, n (%)	22 (22.4%)	40 (33.9%)	91 (91.9%)	<0.0001
Pack/year, n	1.1 ± 4.0	1.72 ± 5.6	32.1 ± 26.7	<0.0001
Passive smoking, yes, n (%)	33 (33.7%)	56 (47.5%)	51 (51.5%)	0.03
Exposure to wood smoke, yes, n (%)	17 (17.3%)	26 (22%)	32 (32.3%)	0.04
Age of wheezing onset, n (%)				–
<5	n/a	7 (5.9%)	0 (0%)	
5-14	n/a	33 (28.0%)	1 (53.5%)	
15-40	n/a	38 (32.2)	8 (52.5%)	
>40	n/a	32 (27.1%)	44 (44.4%)	
ER visits in the last year due to exacerbation of respiratory symptoms	0	1.2 ± 3	0.6 ± 1.1	<0.0001
mMRC	0.2 ± 0.5	1.1 ± 0.9	1.6 ± 1.0	<0.0001
ACQ-5	n/a	4.54 ± 4.4	n/a	–
CAT	n/a	n/a	13.8 ± 7.3	–
Pre-FEV_1%_	96.3 ± 18.4	71.1 ± 20.5	55.3 ± 20.1	<0.0001
Post BD FEV_1%_	98 ± 18.1	77.2 ± 21.9	59 ± 21.1	<0.0001
Pre FEV_1_/FVC, %	80 ± 6	69 ± 12	60 ± 13	<0.0001
Post BD FEV_1_/FVC, %	82 ± 6	71 ± 12	60 ± 13	<0.0001
Blood eosinophils, cells/µl	140 (90–192)	220 (120–312)	190 (130–310)	<0.0001
Blood neutrophils, cells/µl	3090 (2517–3862)	3815 (3122–4940)	4170 (3430–5310)	<0.0001
Blood monocytes, cells/µl	435 (357–525)	500 (390–602)	540 (450–680)	<0.0001
Blood lymphocytes, cells/µl	2185 (1720–2600)	2145 (1670–2642)	1950 (1590–2440)	0.13
Total, IgE, kU/l	57.7 (21-189.7)	134.3 (44.4-370)	71.9 (23.6-175.4)	< 0.0001
IgE to *D. pteronyssinus*	0.04 (0.03-0.21)	0.24 (0.04-6.54)	0.05 (0.03-0.24)	< 0.0001
IgE to *B. tropicalis*	0.02 (0.00-0.11)	0.21 (0.01-2.39)	0.02 (0.00-0.35)	< 0.0001
IgE to *A. lumbricoides*	0.02 (0.00-0.07)	0.05 (0.01-0.36)	0.03 (0.00-0.15)	< 0.0001
Inhaled corticosteroid dose				–
High	0 (0%)	34 (28.8%)	2 (2%)	
Medium	0 (0%)	35 (29.7%)	19 (19.2%)	
Low	0 (0%)	42 (35.6)	5 (5.1%)	
None	0 (0%)	5 (4.2%)	68 (68.7%)	
Charlson comorbidity index	0.05 ± 0.26	1.2 ± 0.6	1.3 ± 0.7	<0.0001
Cardiovascular disease, yes, n (%)	0 (0%)	5 (4.2%)	11 (11.1%)	–
Diabetes, yes, n (%)	1 (1%)	8 (6.8%)	11 (11.1%)	–

BMI, Body Mass Index; ER, emergency room; mMRC, modified Medical Research Council dyspnea scale; ACQ-5, asthma control questionnaire; CAT, COPD Assessment Test; FEV1, forced expiratory volume in 1 second; FVC, forced vital capacity. n/a, not applicable.

### COPD Patients Have Increased Levels of Several Plasma Inflammatory Proteins

We found differences in 41 plasma proteins between patients and healthy controls (Kruskal Wallis test, nominal P < 0.05). Similar results were obtained by the analysis of variance (ANOVA) and 36 proteins remained significant after Benjamini–Hochberg false discovery rate (FDR) correction (**Figure 1B**). The top significant proteins are presented in **Figure 1C**.

**Figure 2:**
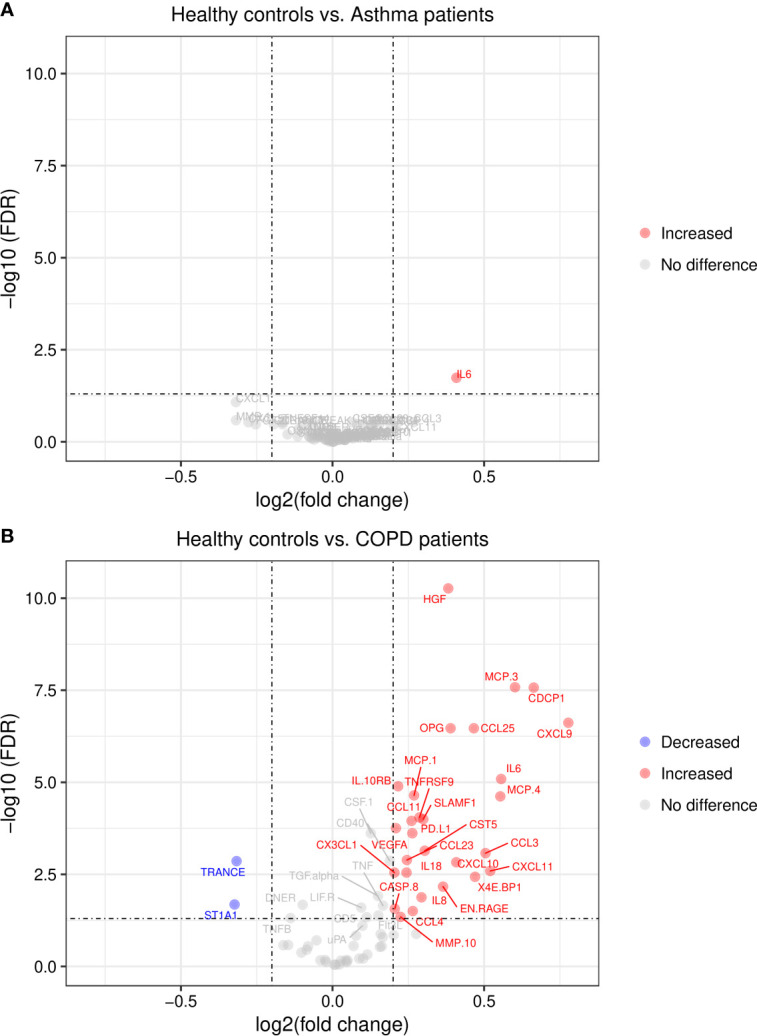
Volcano plots of differentially expressed plasma proteins. **(A)** in asthma patients and **(B)** in COPD patients. Lines indicate cut-off points for FDR < 0.05 and log_2_ fold change ≥0.2; FDR, False Discovery Rate.

We then compared protein levels between each disease group and healthy controls by *t*-test. The levels of 8 proteins showed differences in asthmatic patients (IL-6, CXCL1, MMP-1, CSF-1, CXCL5, CCL3, CCL23 and TNFSF14, nominal *P* < 0.05) while 40 proteins showed significant differences in COPD patients. However, when applying the FDR correction for multiple testing, only IL-6 remained significant in asthma patients (**Figure 2A**) and 39 proteins remained significant in COPD patients (**Figure 2B**).

### Differentially Expressed Proteins Are Grouped in Different Clusters and Are Involved in Recognized Inflammatory Pathways

**Figure 3:**
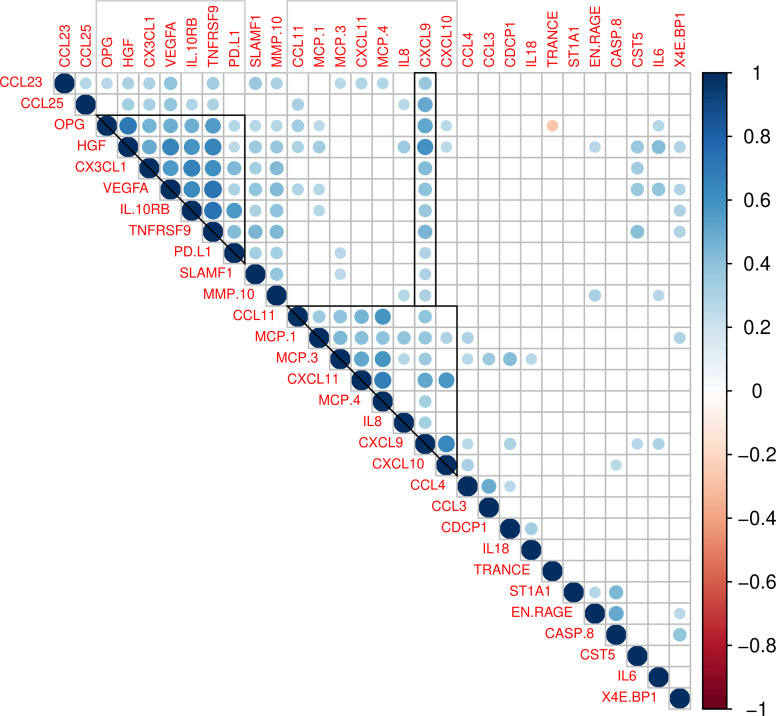
Correlations between plasma levels in 30 proteins showing significant differences between COPD patients and controls (FDR < 0.05 and log_2_ fold change > 0.20). Colored circles indicate a significant correlation (*P* < 0.05). The color scale represents the Pearson correlation coefficient (1 positive correlation, -1 negative correlation). Grey squares indicate clusters of correlated proteins.

We also performed correlation analysis to evaluate the relationships between proteins levels and identify co-regulated signatures in 30 proteins with significant differences in COPD patients (FDR < 0.05 and log_2_ fold change > 0.20). We found two correlation clusters: the first within seven proteins (OPG, HGF, CXC3L1, VEGFA, IL10RB, TNFRSF9, PD-L1) exhibiting high to moderate correlation. The strongest coefficients were observed between TNFRSF9 and VEGFA (r = 0.73, *P* = 1.1 x 10^-17^), TNFRSF9 and IL10RB (r = 0.73, *P* = 9.9x 10^-18^) and HGF and OPG (r = 0.72, *P* = 8.6 x 10^-17^). The second cluster involved eight chemokines with moderate correlation in their protein levels (CCL11, MCP-1, MCP-3, CXCL11, MCP-4, IL-8, CXCL9 and CXCL10). The most significant were CXCL11 and MCP-4 (r = 0.67, *P* = 1.9 x 10^-14^), CXCL9 and CXCL10 (r = 0.65, *P* = 5.1 x 10^-13^), MCP-3 and MCP-4 (r = 0.58, *P* = 2.1 x 10^-10^) and CXCL11 and CXCL10 (r = 0.57, *P* = 5.3 x 10^-10^). These analyses also showed that CXCL9 levels correlate with proteins in the first cluster and TRANCE (RANKL) had an inverse correlation with its ligand OPG (r = −0.27, *P* = 5.5 x 10^-3^) (**Figure 3**).

**Figure 4:**
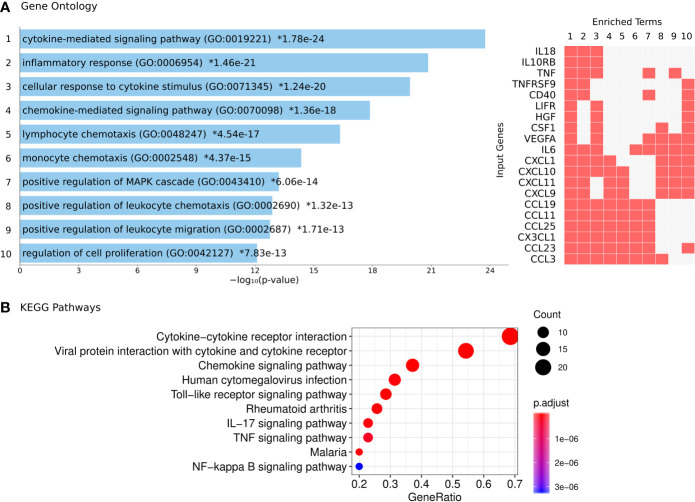
Functional annotation of the differentially expressed plasma proteins in COPD patients. **(A)** Gene Ontology analysis: each bar represents a significant GO term with its *P* value for enrichment. Asterisks indicate significance after correction for multiple testing. The proteins detected in this study that coincide with those involved in each biological process (1 to 10) are indicated by a red box (right panel). **(B)** KEGG pathways with significant enrichment of differentially expressed proteins.

To further evaluate the biological relationships between the differentially expressed proteins in COPD, we performed gene ontology and pathways analyses. Differentially expressed proteins were enriched in the Gene Ontology (GO) categories of cytokine and chemokine signaling pathways (**Figure 4A**). The analysis on KEGG pathways showed the top significant enrichment in the pathways of “cytokine-cytokine receptor interaction”, “Viral protein interaction with cytokine and cytokine receptor”, “chemokine signaling”, “Toll-like receptor signaling “, TNF signaling pathway” and the “NF-kappa B signaling pathway” (**Figure 4B**). The proteins enriched in these biological processes involve several chemokines.

**Figure 5:**
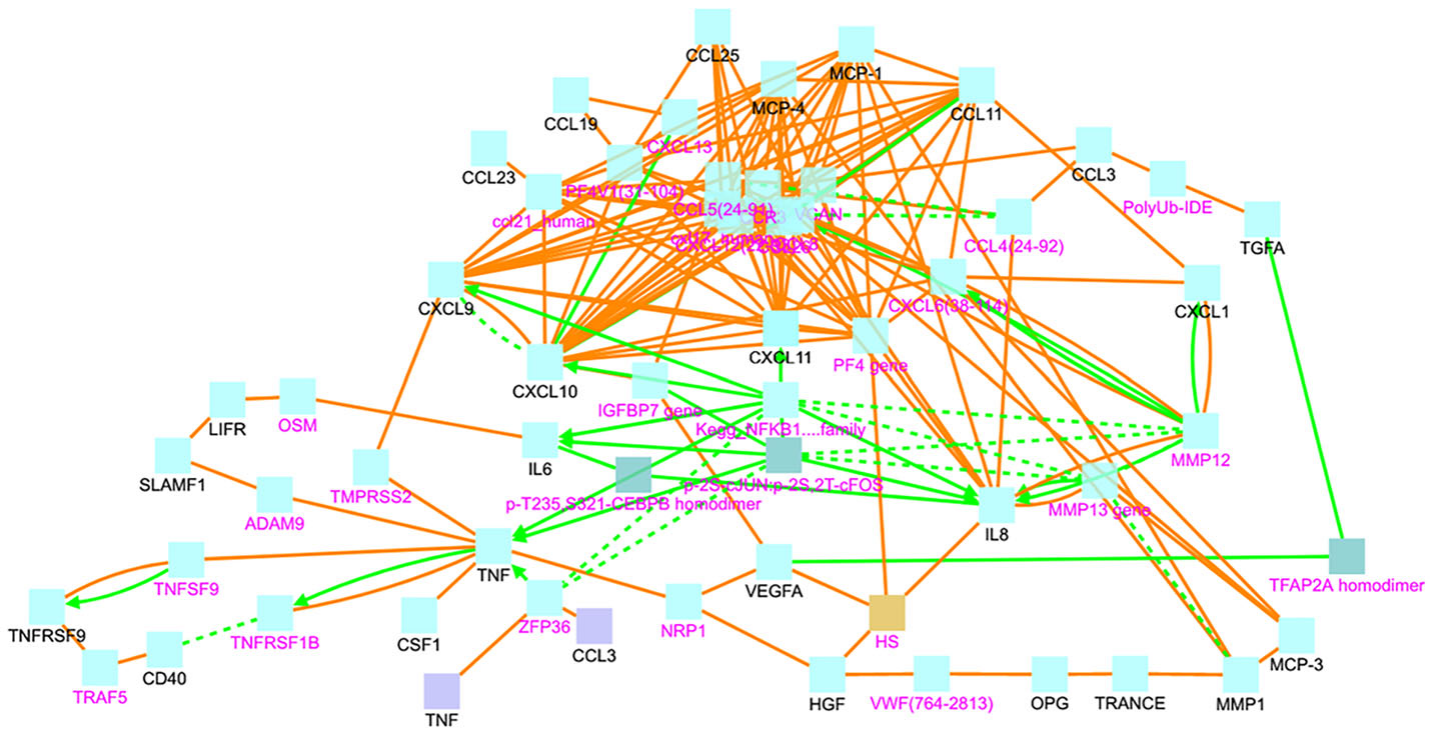
Induced network analysis showing the protein-protein interactions (orange line) and biochemical reactions (green lines) among the differentially expressed proteins (black letters) or mediated by interconnecting nodes (magenta letters). VWF, von Villebrand Factor; HS, heparan sulphate.

In addition, we explored the biological connections among differentially expressed proteins using them as seeds in an induced network analysis considering evidence of binary protein-protein interactions at high level of confidence and biochemical reactions. We found several interactions between the cluster of HGF, OPG, TRANCE and VEGFA with the cluster of chemokines. HGF and OPG have a protein interaction mediated by the von Villebrand factor (VWF). Upon that OPG interacts with its ligand TRANCE, metalloproteinases (MMP) and the chemokine MCP-3. Besides, an interaction mediated by heparan sulphate (HS) links HGF with IL-8 and VEGFA. The interaction of HGF with the chemokine cluster is also mediated by Platelet Factor 4 (PF4). HGF also interacts with TNF involving neuropilin 1 (NRP1) (**Figure 5**).

We also observed an interaction between TNF and CXCL9 that has TMPRSS2 as an intermediate node. From there, CXCL9 and CXCL10 seem to be immune checkpoints connected with several other chemokines. IL-6 and IL-8 are interconnected between them and with the chemokine cluster by members of the NFκB signaling pathway (**Figure 5**).

### Asthma, COPD and Severe COVID-19 Patients Shared Increased Plasma Inflammatory Proteins

**Figure 6:**
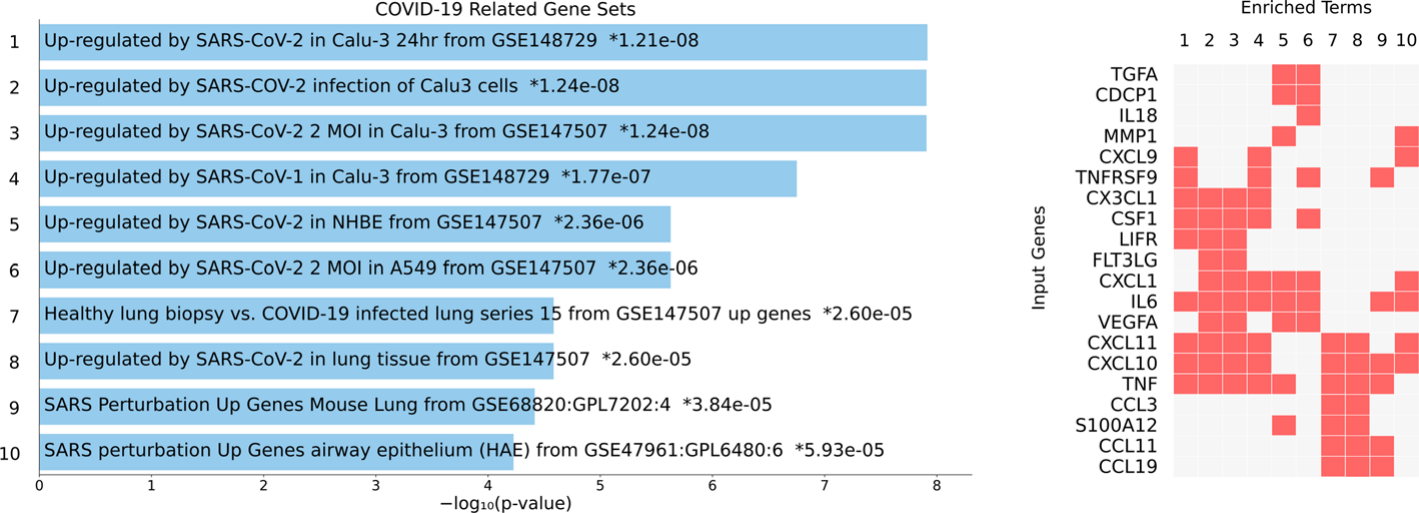
Overlap between genes upregulated upon coronavirus infection with the proteins detected in this study. The details on the experiment are indicated on the blue bars (left) with the *P* value for the enrichment analysis. Asterisks indicate significant enrichment after correction for multiple testing. The proteins detected in this study that coincide with the genes found in each experiment (1 to 10) are indicated by a red box (right panel).

The differentially expressed proteins in COPD were not only enriched in the pathway of viral protein interaction (**Figure 4B**) but were also found enriched in datasets of genes upregulated by SARS coronaviruses. The COVID-19 Drug and Gene Set Library retrieved at least 10 experiments with significant enrichment of the genes encoding the altered proteins detected in this study upon infection. The top significant association was with nine genes upregulated by SARS-CoV-2 in Calu3 cells including CXCL9, TNFRSF9, CX3CL1, CSF1, LIFR, IL6, CXCL11, CXCL10 and TNF (**Figure 6**), suggesting that our findings are supported by gene expression experiments.

**Figure 7:**
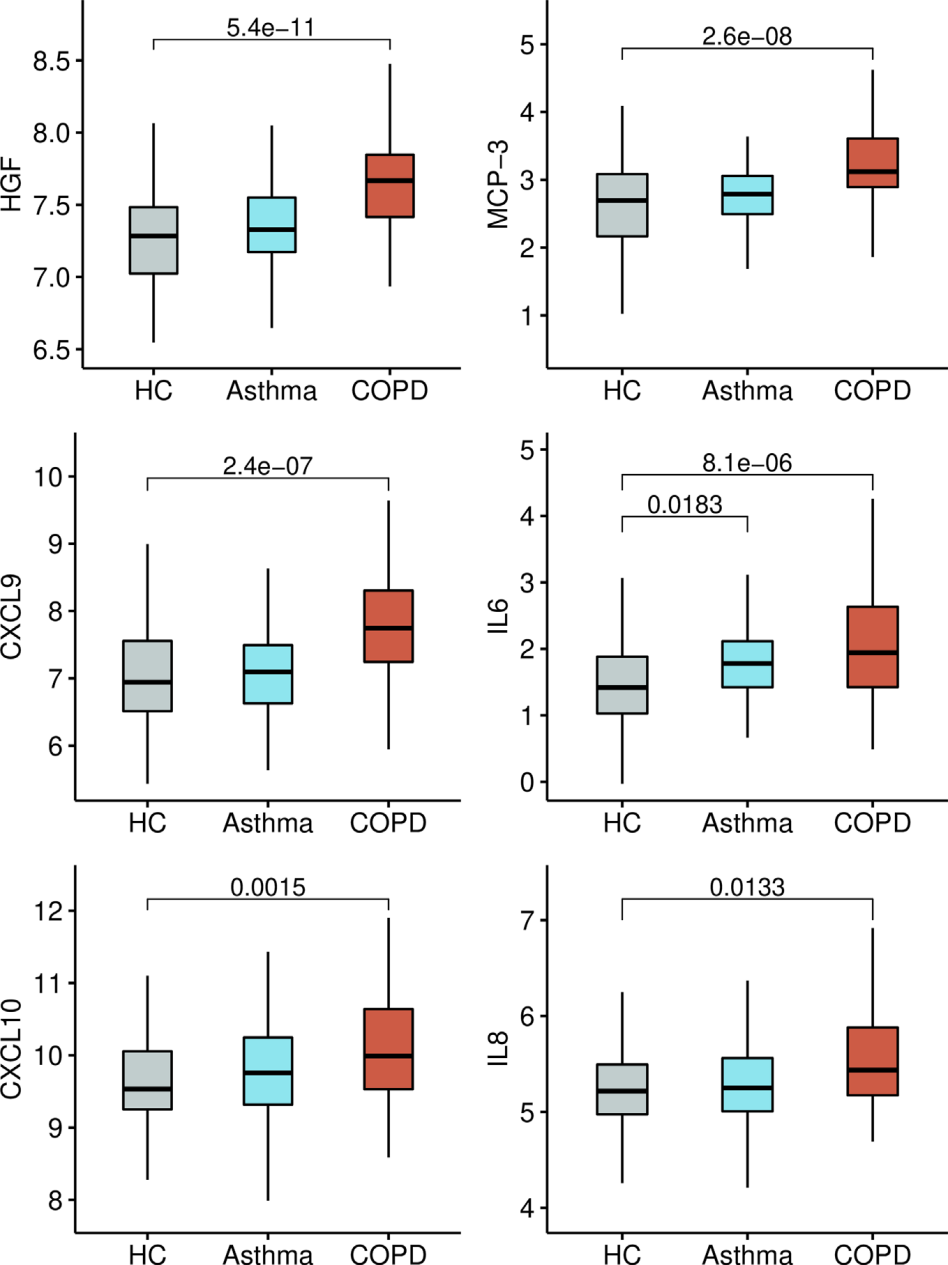
Boxplots with the normalized protein levels (log_2_ scale) of six proteins associated with severe COVID-19 in non-infected healthy controls (HC), asthma and COPD patients.

A comparison with 133 SARS Literature-Associated Genes in Geneshot GeneRIF revealed significant overlap with four proteins found increased in our patients (CXCL10, CXCL9, IL-6, TNF, *P* = 6.9 x 10^-11^). We then analysed which of the differentially expressed proteins detected in this study have been previously reported associated with the severity of SARS-Cov-2 infection in humans. Strikingly, we found that the top significant protein HGF have been reported increased in critically ill COVID-19 patients (17, 26). Also, other proteins such as IL-6, MCP-3, CXCL10, TNF, and EN-RAGE have been found increased in patients with severe COVID-19 (16). The protein levels of eight severe COVID-19 biomarkers (HGF, CXCL9, IL-8, IL-6, MCP-3, VEGFA, CXCL10, CCL3) as detected in healthy controls, asthmatics and COPD patients from this study are presented in **Figure 7**.

### Several Plasma Proteins Associated With COPD Are Influenced by Age and Gender

**Figure 8:**
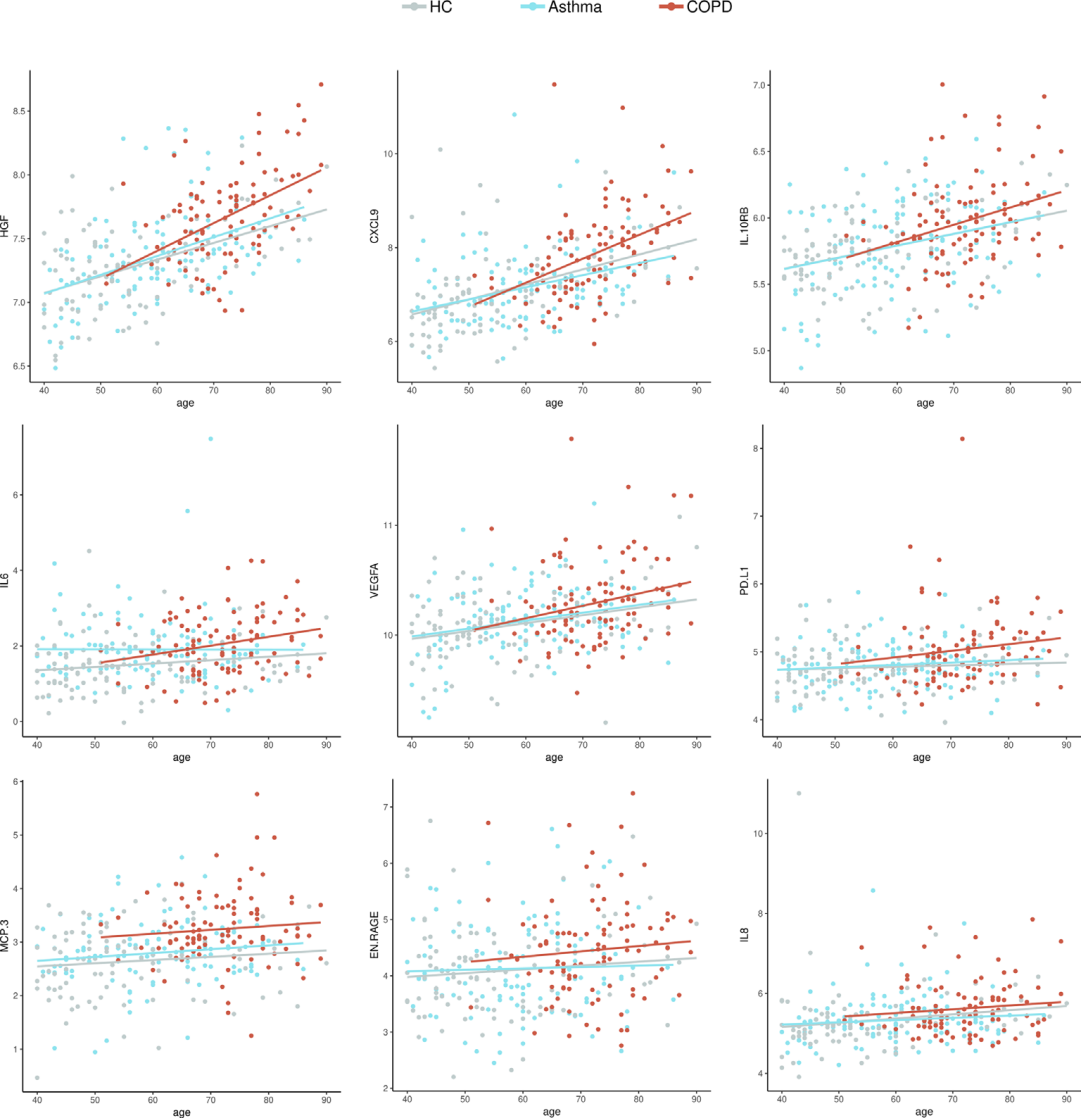
Dot plots with the normalized protein levels (log_2_ scale) in relation to age, each dot represent a subject. Lines represent the regression.

Plasma levels in 18 proteins showed significant correlations with age. The highest coefficients were detected with OPG (r=0.59, *P* = 9.3 x 10^-11^), HGF (r=0.48, *P* = 3.2 x 10^-7^) and CXCL9 (r=0.43, *P* =7.6 x 10^-6^). Of these age-correlated proteins, six had been also associated with COVID-19 severity: HGF and CXCL9 shown the highest coefficients while IL10RB, VEGFA, IL-6 and TNF showed low albeit significant correlation (r < 0.30). Besides, MCP-3, EN-RAGE and IL-8 did not show relation with age. The levels of nine biomarkers of severe COVID-19 according to age and disease group are presented in **Figure 8**. The matrix of correlation coefficients between these biomarkers and age is presented in online **Supplementary Figure 1**.

When comparing the biomarker levels according to gender in the COPD group, IL-6 levels were remarkably higher in females (2.31 ± 0.6) compared to male patients (1.8 ± 0.8) (P=0.008). MCP-3 was higher in males (3.38 ± 0.7) compared to female patients (3.0 ± 0.4) (P=0.007). IL-8 and PD-L1 showed a marginal albeit significant increase in male patients. The rest of the markers (CXCL9, EN-RAGE, HGF, IL10-RB, VEGFA) were similar between female and male patients. In asthmatics, PD-L1 (4.9 ± 0.3 *vs.*. 4.7 ± 0.3, P=0.008) and EN-RAGE (4.4 ± 0.8 *vs.* 3.9 ± 0.7, P=0.003) were significantly increased in male patients compared to female patients.

## Discussion

This study revealed that patients with COPD have significant differences in several plasma proteins compared to healthy controls. Interestingly, some of these proteins, mainly HGF, CXCL9, MCP-3, IL-6, IL-8, CXL10, EN-RAGE, IL10RB, VEGFA, CCL3 and TNF have been also found upregulated in patients with severe COVID-19 (16, 18) and some of them such as HGF postulated as biomarkers for the severe forms of this infection (13, 26, 27). These findings add important information regarding the susceptibility of COPD patients to present severe COVID-19. Asthmatic patients only showed increased levels in IL-6 compared to healthy controls (**Figure 2A**), suggesting that a more systemic inflammation in COPD patients might contribute to their increased risk for severe COVID-19.

HGF was the top significant protein marker in our study. Deng et al., reported that HGF levels above 1,128 pg/ml can discriminate severe from non-severe COVID-19 patients, with an 84.6% sensitivity and 97.9% specificity to classify severe patients (AUC of 90.5%). Also, they report that HGF might significantly increase only when inflammation mounts toward an uncontrolled storm (26). However, our results demonstrate that patients with COPD have a significant elevation of this marker independently of SARS-CoV-2 infection. We also found that HGF levels directly correlated with increasing age in all participants, however by age 60 years this increase become more pronounced in COPD patients (**Figure 8**).

The levels of several differentially expressed proteins between COPD patients and healthy controls were influenced by age. For instance, levels of HGF, osteoprotegerin (OPG), TRANCE and CXCL9 increased in aged healthy groups (**Figure 8** and online **Supplementary Figure 2**). Given the relationship of OPG and TRANCE with osteoclastogenesis (27), our data may reflect protein changes due to aging, but also indicate that the HGF-OPG-TRANCE axis is altered at earlier age-points in COPD patients (online **Supplementary Figure 2**). Besides, other proteins associated to severe COVID-19 such as MCP-3, were found increased in COPD patients independently of age (**Figure 8**). The relevance of these findings on the increased susceptibility of aged COPD patients to severe COVID-19 needs to be further defined.

HGF has been linked to a neutrophil signature that predicts critically ill COVID-19 patients (28). In that study, HGF levels directly correlate with absolute neutrophil counts (rho=0.55, *P* < 0.001) and together with resistin and lipocalin-2 are released from neutrophil granules upon activation. Indeed, HGF and three other neutrophil granule proteins (RETN, LCN2, MMP-8) were not elevated in non-ICU patients compared to controls and were only significantly different in patients who developed critical illness (28).

Our induced network analysis revealed protein-protein interactions between HGF and OPG mediated by the von Willebrand factor (VWF) suggesting a connection of these markers with the coagulation system (**Figure 5**). In the study of Thwaites et al., the von-Willebrand factor A2 was elevated in all hospitalized COVID-19 patients and the elevation was higher in severely affected COVID-19 patients (29). Other studies also report the elevation of VWF as risk factor for severe COVID-19 (30). In this setting, our data support that HGF and OPG could be involved in severe COVID-19 by affecting pathways implicated in coagulopathy and thrombosis (31). Since HGF is increased in chronic inflammatory diseases, further studies are needed to establish whether this protein is elevated in severe COVID-19 because a pre-condition related to the comorbidity, or it increases during the infection and is involved in the pathophysiology of severe COVID-19.

Other cytokines such as IL-6 and TNF-α have been documented in severe COVID-19 (32). Indeed, high serum IL-6, IL-8 and TNF-α levels at the time of hospitalization were strong and independent predictors of patient survival (12). Here we found increased levels of IL-6, IL-8 and TNF in COPD patients. The analysis of protein-protein interactions between the differentially expressed proteins detected in this study, revealed an interaction between TNF and CXCL9 that has the viral activator TMPRSS2 as intermediate node (**Figure 5**). This transmembrane serine protease has been found increased in smokers and in COPD patients (5), and mediates the cleavage of the S protein in SARS-CoV-2, thus is instrumental for viral entry (33). CXCL9 was among the top significant proteins between COPD patients and healthy controls (**Figure 1C**). The consequences of these protein interactions need to be experimentally elucidated, however our results suggest that altered levels of TNF, IL-6 and CXCL9 in COPD patients may be involved in the initial steps that predispose COPD patients to severe COVID-19. CXCL9 is a C-X-C motif chemokine induced by interferon, considered a marker of monocyte/macrophage activation that is implicated in lymphocyte infiltration, Th1 polarization and antiviral immune responses. Several studies have reported increased expression of CXCL9 (34–36), as well as CXCL10 and CXCL11 (16), in severe COVID-19 patients, three co-expressed chemokines that were found increased in the COPD patients in our study (**Figures 2B** and **7**).

Another interesting finding in our COPD patients was the enrichment of increased proteins in chemokine and cytokine networks associated with viral proteins interactions (**Figure 4B**). Respiratory viral infections can trigger acute exacerbations in COPD patients. Different viruses are implicated (*e.g*., rhinovirus, influenza, syncytial respiratory virus and coronavirus), being rhinovirus and coronavirus detected in 35.7% and 25.9% of viral infections occurring during these exacerbations (37). Typically, COPD patients are more susceptible to viral infections; the mechanisms involve dysregulated antiviral function of CD8^+^ T cells *via* the PD-1/PD-L1 axis and an altered production of interferons and chemokines (38). Thus, it is possible that the differences in protein plasma levels and their enrichment in viral related pathways observed in this study are the consequences of previous viral infections that induced lung inflammation and alterations on plasma proteome profile. However, since our COPD patients had not experienced exacerbations in at least eight weeks before sampling, these differences could reflect sub-clinical viral infections, as has been described in patients with stable COPD (39). Another possibility is that the protein dysregulations we detected in these patients reflect an abnormal immune response that could explain their increased susceptibility to a broad spectrum of viral infections including SARS-CoV-2.

This study also reveals differences in the levels of some protein biomarkers between males and females, however IL-6 did not differ according to gender in asthmatic patients suggesting that the increase of IL-6 in this group may be related to their phenotype and may not be confused by the different gender proportions.

The panel of proteins upregulated in COPD and severe Covid-19 that are shown in **Figures 7** and **8** were identified after comparing the proteins detected in this study with those reported in the literature as being increased in adult patients with severe Covid-19 in at least 5 independent studies. To make the literature search as unbiased as possible, we performed a search on all proteins that has been experimentally detected in severe COVID-19 in LitCovid, but we also include some bioinformatic analysis of overrepresentation to calculate the possibility that these observations resulted by chance. We also evaluated the expression levels of these molecules in SARS-Cov-2 infected cells (**Figure 6**). These results have the limitation that Calu3 cells is a lung adenocarcinoma cell line that may not reflect the events occurring in COPD, however, comparative expression of RNA sequencing experiments in lung tissue from COPD patients and healthy controls (E-MTAB-8251, Array Express) also reveals that these molecules are expressed in the lung of COPD patients.

In conclusion, our results show dysregulated inflammatory signatures in the plasma proteome of COPD patients that could be associated with their increased susceptibility to severe COVID-19. Since these proteins were found increased in patients without SARS-CoV-2 infection, they could be further evaluated as biomarkers of increased risk to severe COVID-19. Based on our results, we suggest that measuring levels of HGF, CXCL9, IL10-RB, MCP-3 and EN-RAGE could be further evaluated in larger cohorts as biomarkers of a “pre-morbid state” for the susceptibility to severe COVID-19. The detection of dysregulated plasma proteins in other conditions known to be highly susceptible to severe COVID-19 will provide important information for preventing and treating this viral disease. Moreover, the proteins detected in this study could be analysed as potential targets of new or repositioned therapeutic approaches aiming to avoid severe COVID-19 in patients with COPD.

## Data Availability Statement

The raw data supporting the conclusions of this article will be made available by the authors, without undue reservation.

## Ethics Statement

The studies involving human participants were reviewed and approved by the ethical committees of the University of Cartagena (nr. 4169722017) and the “Fundación Neumológica Colombiana” (nr. 232-07122017). The patients/participants provided their written informed consent to participate in this study.

## Author Contributions

NA and LC: conceived idea for the study. NA, CT-D, RD, and LC: designed the study. NA, JE-G, RR, JR, LF, and CT-D: collected data. NA and HE: performed statistic and bioinformatic analyses. NA, JE-G, HE, and LC: data analyses. LF, RD, CT-D, and LC: provided clinical/primary care expertise on asthma and COPD. NA and LC: drafted the manuscript. All authors contributed to the article and approved the submitted version.

## Funding

This work was financed by the Ministry of Science (Republic of Colombia, Grant 756-2017) and the University of Cartagena. The funders have not role in study design; in the collection, analysis and interpretation of data; in the writing of the report; or in the decision to submit the article for publication.

## Conflict of Interest

The authors declare that the research was conducted in the absence of any commercial or financial relationships that could be construed as a potential conflict of interest.

## Supplementary Material

Supplementary Figure 1 The HGF, OPG and TRANCE axis in patients and controls. **(A)** Schema on the protein-protein interactions between HGF and OPG mediated by fibronectin (FN1), vitronectin (VTN) and the von-Willebrand factor (VWF). **(B)** Dot plots with the normalized protein levels (log_2_ scale) in relation to age, each dot represents a subject. Lines represent the regression.

Supplementary Figure 2 Pearson correlation coefficients of the significant relationships between age and nine proteins associated with severe COVID-19 in healthy controls (HC), asthma and COPD patients.

Supplementary Table 1 Detailed information on the list of plasma proteins with significant differences between COPD patients and healthy controls.

## Glossary

**Table:**

CCL3	C-C motif chemokine ligand 3 or Macrophage inflammatory protein 1-alpha
CCL4	C-C motif chemokine ligand 4 or Macrophage inflammatory protein 1-beta
CCL11	C-C Motif chemokine ligand 11 or eotaxin-1
COPD	Chronic obstructive pulmonary disease
COVID-19	Coronavirus disease 19
CSF-1	Colony stimulating factor 1
CST5	Cystatin-D
CX3CL1	C-X3-C motif chemokine ligand 1 (fractalkine)
CXCL1	C-X-C motif chemokine ligand 1 or Neutrophil-activating protein 3
CXCL5	C-X-C motif chemokine ligand 5 or Neutrophil-activating protein 78
CXCL9	C-X-C motif chemokine ligand 9 or Monokine induced by interferon-gamma
CXCL10	C-X-C motif chemokine ligand 10 or IP-10
CXCL11	C-X-C motif chemokine ligand 11 or interferon-inducible T-cell alpha chemoattractant
EN-RAGE	extracellular newly identified receptor for advanced glycation end-products binding protein or S100 Calcium Binding Protein A12
HGF	Hepatocyte Growth Factor or lung fibroblast-derived mitogen
IL10RB	Interleukin 10 receptor subunit beta
KEGG	Kyoto Encyclopedia of Genes and Genomes
MCP-3	Monocyte chemoattractant protein 3 or CCL7
MCP-4	Monocyte chemotactic protein 4 or CCL13
MMP-1	Matrix metallopeptidase 1
OPG	Osteoprotegerin or Tumor necrosis factor receptor superfamily, member 11b
SARS-CoV-2	Severe acute respiratory syndrome coronavirus 2
SLAF1	Signaling lymphocytic activation molecule family member 1
ST1A1	Sulfotransferase family 1A member 1
TMPRSS2	Transmembrane serine protease 2
TNF	Tumor Necrosis Factor
TNFRSF9	TNF receptor superfamily member 9
TNFSF14	TNF superfamily member 14
TRANCE	TNF superfamily member 11 or Receptor activator of Nuclear Factor Kappa B Ligand
VEGFA	Vascular Endothelial Growth Factor A
