# Supplementary figures and images for: Chronic Obstructive Pulmonary Disease Patients Have Increased Levels of Plasma Inflammatory Mediators Reported Upregulated in Severe COVID-19
Source: Front Immunol. 2021 Jul 15;12:678661. doi: 10.3389/fimmu.2021.678661 (PMC8320593; doi:10.3389/fimmu.2021.678661)

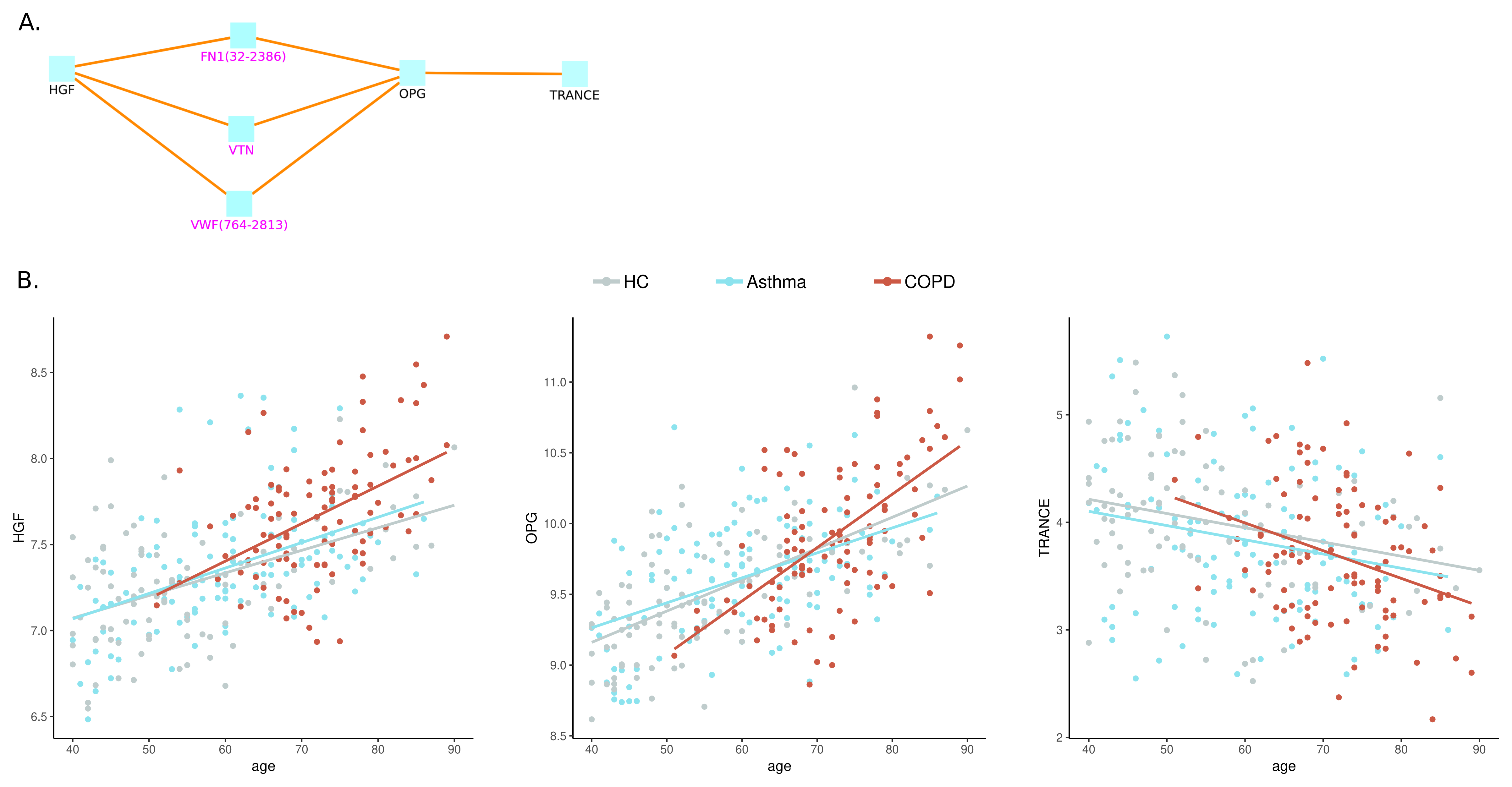

Supplement: Supplementary Figure 1 — The HGF, OPG and TRANCE axis in patients and controls. (A) Schema on the protein-protein interactions between HGF and OPG mediated by fibronectin (FN1), vitronectin (VTN) and the von-Willebrand factor (VWF). (B) Dot plots with the normalized protein levels (log2 scale) in relation to age, each dot represents a subject. Lines represent the regression. [file Image_1.png]

age

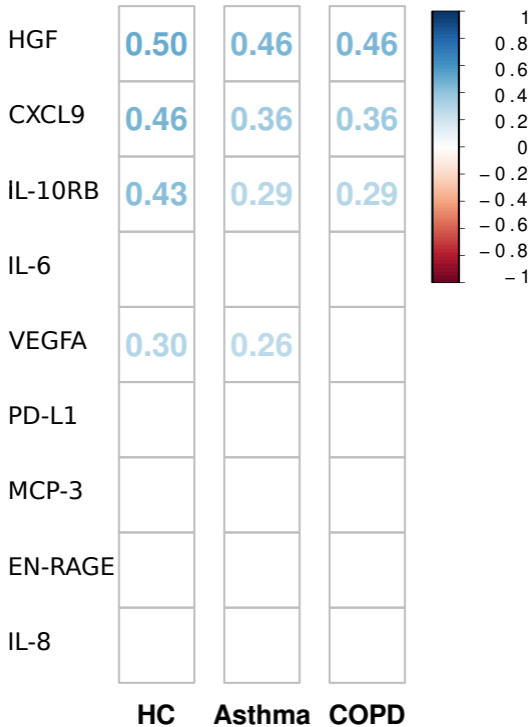

Supplement: Supplementary Figure 2 — Pearson correlation coefficients of the significant relationships between age and nine proteins associated with severe COVID-19 in healthy controls (HC), asthma and COPD patients. [file DataSheet_1.pdf]
